# A Case of Isolated Dysarthria in a COVID-19 Infected Stroke Patient: A Nondisabling Neurological Symptom With Grave Prognosis

**DOI:** 10.7759/cureus.9921

**Published:** 2020-08-21

**Authors:** Riwaj Bhagat, Siddharth Narayanan, Bibodh J Karki, Wei Liu, Kerri Remmel

**Affiliations:** 1 Neurology, University of Louisville, Louisville, USA; 2 Surgery, University of Louisville, Louisville, USA; 3 Infectious Disease, University of Louisville, Louisville, USA

**Keywords:** covid-19, stroke, dysarthria, atrial fibrillation

## Abstract

Isolated dysarthria is a speech abnormality characterized by slurring without any language dysfunction, or other neurological deficits. In an acute setting, it is commonly associated with stroke. In the context of social distancing during the current corona virus disease 2019 (COVID-19) pandemic, nondisabling symptoms such as isolated dysarthria can delay a patient's perception to seek immediate medical care. We present a rare case of isolated dysarthria in a COVID-19 infected stroke patient with a grave prognosis. A 79-year-old African American male presented with isolated dysarthria that manifested two days prior to his hospital visit. The dysarthria assessment showed impaired articulation, phonation, and prosody. Other neurological examinations were normal. He tested positive for the COVID-19 infection. His pulmonary CT scan showed bilateral ground glass opacities. An electrocardiogram showed atrial fibrillation (AF). Brain MRI revealed a punctate acute infarction in the left frontal lobe. Initially, he was treated with IV anticoagulation, oral beta-blocker, azithromycin and hydroxychloroquine, but he dramatically deteriorated within a week exhibiting a highly elevated cytokine level eventually resulting in multi-system organ failure. Despite aggressive treatment with steroids, tocilizumab and other supportive measures, the patient died of cardiac arrest. Our case highlights that acute stroke could manifest as an isolated dysarthria, which is an indicator of increased severity and high mortality with COVID-19 infection. Public awareness about the stroke symptom awareness should be emphasized.

## Introduction

As per the World Health Organization, corona virus disease 2019 (COVID-19) pandemic caused by the novel severe acute respiratory syndrome coronavirus 2 (SARS-CoV-2) has resulted in more than half million deaths, as of July 2020 [1]. Various neurological manifestations have been associated with patients having COVID-19 infection, among which headache and anosmia have been identified as common early features [2]. Acute stroke has been reported both in the early and late phase of the disease [3-5]. Acute disabling neurological deficits such as limb weakness, numbness, and vision loss usually alarm the patient or their family members to seek immediate medical attention. Nondisabling symptom like isolated dysarthria, defined as abnormalities in speed, strength, steadiness, range, tone, or accuracy of movements required for the control of speech without evidence of any language impairment or other neurological deficits [6], may be assumed as nonemergency medical situation by the general population who are unaware of the stroke symptoms delaying an urgent medical care [7]. We present a case of isolated dysarthria in a COVID-19 infected stroke patient associated with new-onset atrial fibrillation (AF), whose reluctance to seek immediate medical care in the context of social distancing resulted with a grave prognosis.

## Case presentation

A 79-year-old African American male with a past medical history of hypertension presented with a static slurred speech that manifested with sudden onset two days prior to his hospital visit in late January of 2020. Following the slurred speech the next day he had chills, however, did not record his body temperature at home. He initially considered his symptoms as a minor flu, however, later decided to seek medical care due to nonresolving speech impairment. On presentation, he was febrile with an oral temperature of 100oF. His blood pressure was 124/74 mmHg. He endorsed compliance of his antihypertensive medication. He did not give a history of tobacco abuse, shortness of breath, cough, or intermittent palpitation. Physical examination showed an irregular pulse at the rate of 96 beats/min, respiratory rate at 18 breaths/min, and fine crackles in bilateral lower lungs on auscultation. His blood oxygen saturation level was 95%. Neurological examination was unremarkable except mild dysarthria. From his dysarthria assessment it was inferred that he had a low pitched phonation, impaired prosody for the sentence “ I am hungry”, lack of hypo- or hyper-nasality, decreased rate of repetitions (repeated the word “buttercup” only four times in five seconds), impaired articulation of the word “PresbyEpiscopal” and letters “P, B, F”, but with normal articulation of the words “la-la-la”. The initial National Institute of Health Stroke Scale (NIHSS) was one.

**Figure 1:**
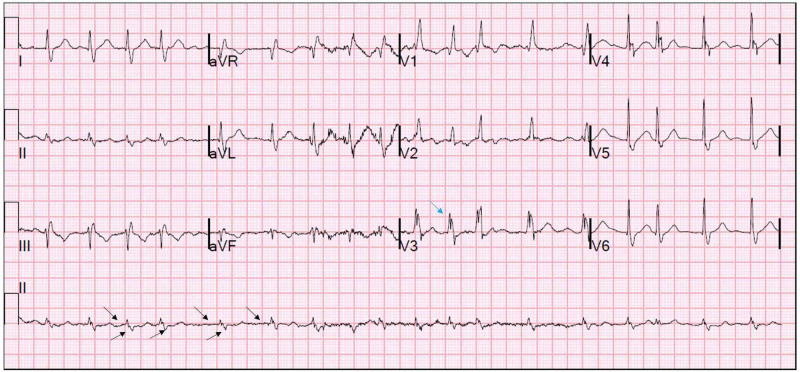
Electrocardiogram. A 12-lead electrocardiogram showing AF (black arrow). The ventricular rate was 108 bpm with a right bundle branch block (blue arrow). AF, atrial fibrillation

The real-time reverse transcription polymerase chain reaction (RT-PCR) analysis of SARS-CoV-2 infection tested positive from the nasopharyngeal swab. Pertinent initial laboratory findings showed a white blood cell count of 9.3 (4.5-11 K/μL), lymphocyte count of 0.6 (1.2-3.4 K/μL), and giant platelet morphology with normal count. He had mild elevated creatinine 2.4 (0.7-1.5 mg/dL) and procalcitonin levels 0.19 (0.02-0.10 ng/mL). His interleukin-6 92 (0-6.3 pg/mL), ferritin 505 (15-150 ng/L), and D-dimer 1201 (0-230 ng/mL) levels were abnormal. His cardiac troponin levels were normal, but the transaminase levels were elevated. The electrocardiogram showed an AF with a rapid ventricular response (Figure 1).

**Figure 2:**
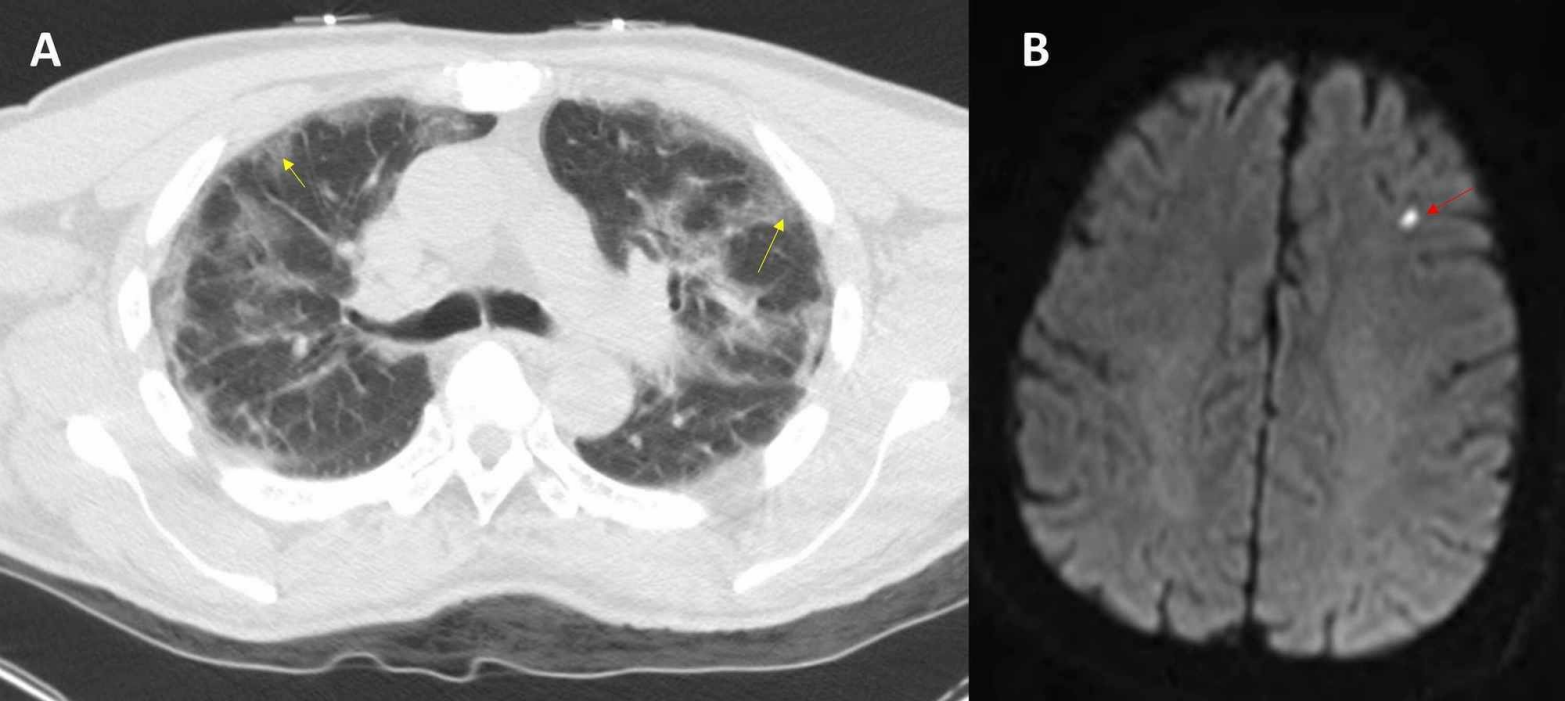
CT of the chest and MRI of the brain. A: CT of the chest showing bilateral peripheral ground glass opacities (yellow arrow). B: MRI of the brain showing an acute infarct in the left frontal lobe (red arrow).

The CHA2DS2-VASc score was four (calculated from his risk factors: age, hypertension, and stroke). A pulmonary CT scan showed bilateral peripheral ground glass opacities without pulmonary embolism (Figure 2A), and a brain MRI showed a punctate left frontal lobe infarction (Figure 2B). 

**Figure 3:**
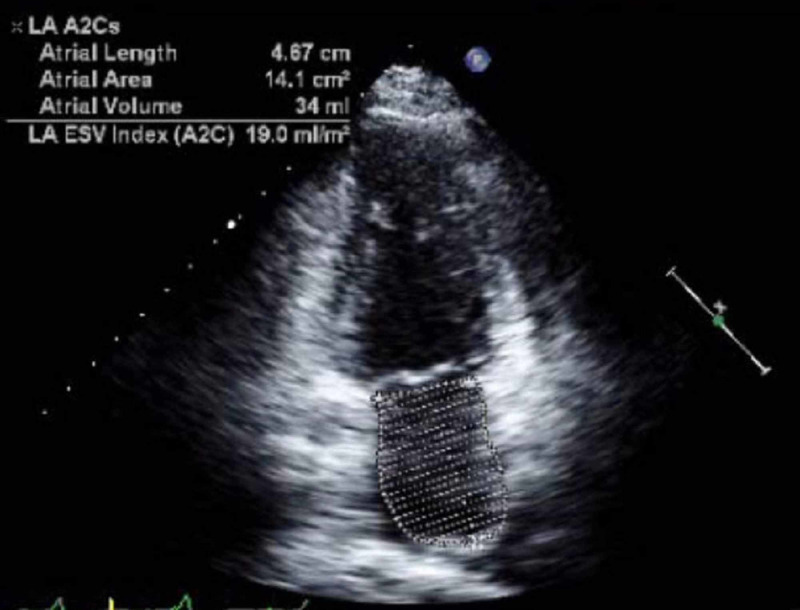
Echocardiogram. Dedicated apical two chamber view of left atrium and left ventricle shows moderate left atrial enlargement as evidenced on modified biplane method with calculated volume of 34 mL/m^2^

A magnetic resonance angiogram of the head and neck showed no significant stenosis and transthoracic echocardiogram showed normal systolic function, moderate left atrial enlargement without thrombus or atrial septal defect (Figure 3). 

The patient was treated with IV unfractionized heparin infusion at the rate of 15 units/kg/hour and metoprolol tartrate 25 mg two times a day. The COVID-19-associated pneumonia was treated with oral azithromycin and hydroxychloroquine and was closely monitored in the ICU setting. Still, after a week, the patient got an increased respiratory distress as a consequence of a cytokine storm and required mechanical ventilation. The laboratory findings during this time showed highly abnormal interleukin-6 330 (0-6.3 pg/mL) level. The patient was treated with IV steroids and tocilizumab, but his condition further deteriorated, complicated by a multi-system organ failure, including septic shock, acute respiratory distress syndrome, acute myocardial injury (NT-proBNP 936 pg/mL), acute kidney injury, and dyselectrolemia. Despite aggressive supportive measures that included hemodialysis, the patient died two and half weeks after his admission. 

## Discussion

Based on our literature search, we believe this is the first report to identify an isolated dysarthria as a presenting symptom in a COVID-19 infected stroke patient. Acute stroke in COVID-19 patients is commonly reported in severe cases and during the later course of the disease [3]. However, stroke as an early manifestation from COVID-19 is less common and has been mainly reported with large vessel occlusion of anterior circulation [4-5]. Usually dysarthria in a COVID-19 infected stroke patient is clustered with other neurological deficits [8].

Isolated dysarthria is an uncommon presentation often associated with transient ischemic attack (TIA) and acute stroke. Other causes include cranial neuropathies, acute metabolic disturbances, and inflammatory diseases [9]. Dysarthria can be assessed by various dysarthria assessment tools among which the Frenchay Dysarthria Assessment is a standardized tool commonly used by the speech-language pathologist [10]. In general, strength, volume, speed, vocal quality, tone, breath control, pitch, range, and steadiness of speech are assessed. These relate to articulation, phonation, respiration, nasality and prosody, and affect intelligibility, audibility, and efficiency of spoken communication. Based on these assessments, dysarthria is then classified as flaccid, spastic, ataxic, hypo- or hyperkinetic, or the mixed type as a result of various upper or lower motor neurons, or neuromuscular pathologies, or a combination of the two. It is also distinguished from apraxia of speech, a motor planning or programming disorder [6, 11]. Despite the nonspecific topographic localization of isolated dysarthria, the lacunar stroke has been commonly associated with its occurrence [12]. Miller Fisher classification of the lacunar syndrome that included dysarthria were pure motor hemiparesis, ataxic hemiparesis, dysarthria-clumsy hand syndrome, and pure dysarthria [13]. However, dysarthria as such has no clinical localizing value [11]. Standardized dysarthria assessment using the FDA tool was not performed in our case due to the COVID-19 isolation protocol and the patient’s condition. We inferred that our patient likely had a flaccid type of isolated dysarthria related to minimal orofacial weakness, acute in onset, without other identifiable neurological deficits resulting from punctate left frontal lobe stroke. Left hemispheric lesion, independent from lesion topography, has been associated with frequent occurrence and severity of dysarthria [14].

As per the pathophysiology of stroke with COVID-19 infection, various mechanisms involving coagulopathies, changes in lipid metabolism and platelet aggregation, alterations in vascular endothelial function, and plaque instability and rupture have been described [15]. In addition, there is also the potential risk of cardioembolic stroke of subsequent cardiac dysfunction. Various underlying mechanisms for cardiac arrhythmias in COVID-19 patients have been proposed and include the occurrence of a cytokine storm, direct tissue damage, electrolyte abnormalities, hypoxia, and sepsis [16]. AF is the most common arrhythmia noted among elderly individuals [17]. Although, earlier new onset AF is rare [18], it helps predict disease severity and mortality [16, 18].

Even though our patient was given anticoagulation therapy, given a CHA2DS2-VASc score of four, patients with COVID-19 may be prone to increased thromboembolic risk given elevated D-dimer levels, which should be considered during the decision-making regarding anticoagulation initiation for AF, even in the presence of a low CHA2DS2-VASc score [19].

Notably, during this pandemic, there has been a decline in the hospital admission for patients with stroke and TIA given the implementation of social distancing and fear of contracting COVID-19 in a hospital setting. As such, untreated or undiagnosed stroke or TIA can lead to a poor prognosis [20].

In our case, the likely cause of isolated dysarthria was a punctate left frontal stroke. It is difficult to postulate whether the stroke resulted from a cardioembolic event from a paraoxymal AF given normal troponin at the presentation, or from the hypercoagulability and vascular endothelial dysfunction associated with COVID-19 infection. As acute stroke and new-onset AF, both are indicators of increased severity and mortality, isolated dysarthria as such must not be ignored.

## Conclusions

Our case highlights that with COVID-19 infection, isolated dysarthria could be the only neurological manifestation of an acute ischemic stroke. Minor or nondisabling stroke symptoms should not be considered benign delaying the hospital presentation, as stroke is an indicator of increased severity and high mortality with COVID-19 infection and public awareness about stroke recognition should be emphasized. 
